# Signaling architecture of the glucagon-like peptide-1 receptor

**DOI:** 10.1172/JCI194752

**Published:** 2026-01-16

**Authors:** Gregory Austin, Alejandra Tomas

**Affiliations:** Section of Cell Biology and Functional Genomics, Department of Metabolism, Digestion and Reproduction, Imperial College London, London, United Kingdom.

## Abstract

The glucagon-like peptide-1 receptor (GLP-1R) is a class B1 G protein–coupled receptor and major therapeutic target in type 2 diabetes and obesity. Beyond its canonical role in Gα_s_/cAMP signaling, GLP-1R is increasingly recognized as an organizer of spatiotemporally defined signaling nanodomains, or “signalosomes.” This Review highlights our current knowledge on the mechanisms of assembly and regulation of GLP-1R signalosomes, including the involvement of biomolecular condensates formed by liquid-liquid phase separation, and the role of membrane contact sites between the endoplasmic reticulum (ER) and other organelles as key locations for GLP-1R signaling assemblies. Furthermore, we discuss existing data on the molecular composition and functional impact of two predicted GLP-1R nanodomains, one at ER–plasma membrane contact sites, where GLP-1R might interact with ion channels and transporters to influence local excitability and coordinated insulin secretion, and another at ER–mitochondria membrane contact sites, with the capacity to control lipid and calcium signaling and modulate ER and/or mitochondrial activity. We additionally discuss the role of GLP-1R posttranslational modifications as critical modulators of GLP-1R signal specification and nanodomain organization. Conceptualizing GLP-1R as a dynamic architect of spatiotemporally encoded signalosomes opens new avenues for a deeper understanding of incretin biology with the potential for identification of novel GLP-1R effectors and the development of refined therapeutic strategies for metabolic disease.

## Glucagon-like peptide-1 receptor expression and signaling profiles

The glucagon-like peptide-1 receptor (GLP-1R) belongs to the glucagon family of class B1 G protein–coupled receptors (GPCRs), also comprising the glucagon receptor (GCGR), the glucagon-like peptide-2 receptor (GLP-2R), and the glucose-dependent insulinotropic polypeptide receptor (GIPR) (1). Receptors from this family feature a characteristically large (~120 amino acid) extracellular domain that mediates a two-step binding mechanism with their cognate peptide ligands (2). Pharmacological agonists targeting this family of receptors, particularly GLP-1R, have revolutionized type 2 diabetes (T2D) and obesity treatment, and further roles are emerging in the treatment of other related disorders such as metabolic dysfunction–associated steatotic liver disease (MASLD) (3).

GLP-1R is predominantly expressed in pancreatic β cells, where it potentiates glucose-stimulated insulin secretion and promotes β cell survival (4). Expression is also documented in other pancreatic endocrine cell subtypes such as δ cells (5), with some evidence of α cell–localized GLP-1R action (6). Beyond the pancreas, GLP-1R is found in diverse brain regions such as the hindbrain, mesolimbic regions, and brain hypothalamic centers, where it contributes to appetite regulation (7); the heart sinoatrial node, where it might play a direct chronotropic effect (8); vascular smooth muscle cells of the renal afferent arteriole, where it promotes vasodilation, glomerular filtration rate, and natriuresis (9); and macrophages, where it might regulate lipid metabolism (10) and possibly play a direct antiinflammatory role (11).

GLP-1R primarily couples to Gα__s__ proteins to stimulate the synthesis of cyclic 3′-5′-adenosine monophosphate (cAMP) via adenylate cyclase (AC) activation, with its signaling modulated by receptor activity–modifying proteins (RAMPs), G protein–coupled receptor kinases (GRKs), β-arrestins, phosphodiesterases (PDEs), and posttranslational modifications (PTMs) (12–14), with each of these processes well described in other reviews (15–18). Importantly, as for other GPCRs, GLP-1R signaling is not unstructured but occurs within spatially and temporally defined nanodomains (19, 20) that allow controlled activation of signaling effectors such as protein kinase A (PKA), exchange protein activated by cAMP 2 (Epac2), and cAMP response element–binding protein (CREB) (21, 22). The location and organization of these nanodomains, also referred to as “signalosomes,” is predicted to influence GLP-1R signaling outcomes by governing the activation of specific downstream targets, so that direct molecular interaction between receptors, effectors, and targets becomes a key determinant for efficient and coordinated signal transduction. In this Review, we explore our current understanding of the spatiotemporal regulation of GLP-1R signaling, elaborating on the nature of GLP-1R signalosomes and the potential prediction of novel GLP-1R nanodomain components by the spatial resolution of GLP-1R–protein interactions.

## Spatiotemporal compartmentalization of GLP-1R signaling

Stimulation of GLP-1R at the plasma membrane results in a conformational shift in the receptor linked to the activation of heterotrimeric G proteins (23). Specifically, ligand binding leads to outward pivoting of the receptor transmembrane helix 6 (24), enabling a collision-coupling event that activates the G__s__ protein α subunit (Gα__s__), dissociating it from the βγ subunits and allowing it to activate AC (25). There are 9 known membrane-bound AC isoforms (AC1–AC9) that can be activated downstream of Gα__s__ (12), with some studies suggesting the involvement of AC8 in GLP-1R–induced β cell signaling (26). In addition, soluble AC (sAC), also known as AC10, is found within the cytosol and in mitochondria and endosomal lumens, and its activity is further modulated by bicarbonate and Ca^^2+^^ ions (27, 28).

Signaling from GLP-1R is organized in receptor-associated independent nanodomains (RAiNs), concentrated areas of cAMP accumulation that improve signal fidelity. Anton et al. (21) showed that these nanodomains (~60 nm in size) can preserve localized signaling at low ligand concentrations, but fuse into bulk pools under high levels of agonist exposure. cAMP functions at RAiNs as a secondary messenger, activating the downstream effectors Epac2 and PKA. The main kinase in charge of the immediate signal processing of GLP-1R, PKA, is responsible for a plethora of critical processes, including modulation of mitochondrial function and stimulus-induced vesicular secretion (29). Dysregulation of PKA signaling is associated with metabolic dysfunctions such as obesity (30) and T2D (31), mental health disorders such as bipolar disorder (BD) and schizophrenia (32), and neurodegenerative disorders such as Alzheimer’s (33, 34) and Parkinson’s diseases (35).

PKA is a tetrameric enzyme, consisting of two regulatory and two catalytic subunits. The regulatory subunits require stepwise binding of two molecules of cAMP to activate the catalytic subunits, which then phosphorylate their downstream targets (36). The mechanism of activation of the PKA catalytic subunits is still debated. The canonical model suggests that these dissociate from the regulatory subunits upon cAMP binding (37); however, Smith et al. (38) showed that at physiological cAMP concentrations the PKA holoenzyme does not dissociate. This study focused only on PKA type II regulatory (RII) subunits, suggesting potentially different signaling mechanisms depending on the type of PKA regulatory isoform involved. PKA regulatory subunits contain a dimerization and docking domain recognized by A-kinase anchoring proteins (AKAPs), a family of more than 50 signaling scaffold proteins in charge of tethering PKA to specific subcellular locations. This tethering allows localized PKA signaling from defined cellular regions (39), and therefore AKAPs are indispensable components of any receptor cAMP/PKA signaling nanodomain.

While PDEs have been classically considered the primary means of enforcing cAMP nanodomain formation by restricting cAMP diffusion, their catalytic rates alone are insufficient to account for the degree of compartmentalization observed experimentally (18). Recent evidence reveals an alternative mechanism by which the PKA type Iα and -β regulatory subunits (RIα/β) undergo liquid-liquid phase separation (LLPS) to form biomolecular condensates, which act as dynamic cAMP buffers, directly contributing to intracellular cAMP compartmentalization (40, 41). PKA RIα/β condensates are enriched in both cAMP and PKA catalytic activity and are essential for the function of local PDE-based cAMP sinks. In neurons, PKA RIα/β condensates couple excitation to Ca^^2+^^-activated PKA signaling (42), and defects in this process are associated with the development of neurological disorders such as BD and schizophrenia (43, 44), dementia-like disorders (41), and cancer (40). PKA RIα biomolecular condensates have also been shown to play a key role in the regulation of GLP-1R signaling in pancreatic β cells, with their disruption leading to changes in β cell Ca^^2+^^ and cAMP oscillation frequencies, insulin secretion, and CREB-mediated gene expression downstream of receptor activation (45). Building on this, Hardy et al. (46) refined the model of phase separation of PKA signaling by mapping the molecular determinants of PKA RIα condensation. They showed that both the PKA RIα intrinsically disordered N-terminal linker and the β4-β5 loop are essential for the formation of LLPS condensates, and that these structures are dynamically regulated by the phosphorylation status of RIα and its interaction with the PKA catalytic subunits, which together act as molecular switches to control condensate assembly and/or disassembly in response to cAMP signaling dynamics. Moreover, Zaccolo et al. (19, 47) proposed that RIα condensates may serve not only as dynamic buffers that constrain cAMP diffusion, but also as privileged microenvironments where other key components of the cAMP/PKA pathway, such as catalytic subunits, PDEs, AKAPs, and Epacs, as well as downstream targets for PKA phosphorylation, are co-recruited, enhancing the efficiency and spatial precision of assembly of these multiprotein complexes. Thus, these condensates have the potential to play a key role in defining both endogenous GLP-1R effects and clinical responses to GLP-1R agonists. To these emerging concepts, we must add the recent notion that β-arrestins themselves also undergo LLPS to form condensates that regulate GPCR function (48), suggesting a complex mode of GLP-1R condensate assembly and regulation that is potentially further fine-tuned by receptor PTMs (49, 50), as discussed below.

These important studies of GPCR condensate formation and its effects on receptor function have mainly been performed in heterologous systems and only recently started to focus on GLP-1R. Further work is therefore needed to determine the role of LLPS and biomolecular condensate formation on GLP-1R signal organization, as well as to investigate how the subcellular localization and composition of these multimeric assemblies defines the signaling architecture of GLP-1R in physiologically relevant cells with endogenous levels of receptor expression.

## Spatially localized GLP-1R complexomes define novel signaling targets

Protein-protein interactions are central to the regulation of cell signaling, enabling receptors and their effectors to form dynamic, context-dependent macromolecular signaling complexes. In many cases, signaling specificity arises not solely from receptor activation, but from the spatial and temporal organization of these multimeric interactions within the cell. Signaling from GLP-1R is regulated by well-studied interactions with RAMPs (13, 15, 51), β-arrestins (1, 16), and the endosomal sorting machinery (52), which modulate receptor trafficking and signal duration, shaping downstream responses. As interest grows in the spatiotemporal dimension of GPCR signaling, the field of spatial interactomics, including the use of novel time-resolved approaches (53), is beginning to reveal new principles by which the local organization of protein complexes defines signaling outcomes for GPCRs, including GLP-1R.

In this space, four independent proteomic approaches to resolve GLP-1R signaling regulators and downstream targets have converged on a core set of active GLP-1R interactors, despite notable methodological differences (Table 1). Austin et al. (54) and Zhang et al. (55) both employed coimmunoprecipitation of agonist-stimulated FLAG-tagged human GLP-1R coupled with mass spectrometry, performed in the pancreatic β cell lines INS-1 832/3 and MIN6, respectively, yielding highly overlapping datasets that emphasize reproducible interactors within detergent-stable complexes. By contrast, Dang et al. (56) used a GLP-1–APEX2–based proximity labeling strategy in INS-1E β cells and the GLP-1R–expressing human neuronal cell line SK-N-SH, specifically capturing plasma membrane–proximal interacting proteins in the native receptor environment. Several interactors detected by this approach overlapped with those from the previous two coimmunoprecipitation assays, providing orthogonal validation. Finally, Huang et al. (57) applied a split ubiquitin yeast two-hybrid screen, enriching for integral membrane partners less accessible to biochemical or proximity labeling methods.

Among the most striking findings is the repeated identification of ATP1B1, the β1 subunit of the Na^^+^^/K^^+^^-ATPase, as an active GLP-1R interactor in three of the four interactome datasets. The Na^^+^^/K^^+^^-ATPase is a ubiquitously expressed P-type ATPase responsible for the active transport of three Na^^+^^ ions out and two K^^+^^ ions into the cell, using the energy from ATP hydrolysis (58). Structurally, the pump works as a heterotrimeric complex composed of a catalytic α subunit, a regulatory β subunit, and, in many tissues, a small FXYD accessory protein (59). The β1 isoform of the β subunit acts as a chaperone, stabilizing and correctly folding the α subunit, while also influencing pump kinetics and trafficking (60). Briant et al. (61) demonstrated that fatty acid oxidation (FAO) in pancreatic α cells provides ATP to sustain Na^^+^^/K^^+^^-ATPase activity, essential for maintaining membrane action potential amplitude during low-glucose states. When FAO is inhibited, the pump activity is compromised, leading to membrane depolarization and reduced glucagon secretion. Inhibition of the Na^^+^^/K^^+^^-ATPase phenocopied this effect, confirming the central role of this pump in supporting α cell excitability and secretory function under fasting conditions. Interactome data indicate that agonist-stimulated GLP-1R forms a protein-protein interaction with ATP1B1 in β cells, suggesting a relevant role for the receptor in regulating Na^^+^^/K^^+^^-ATPase activity in these cells. Thus, ATP1B1 may represent an important downstream signaling target for GLP-1R, which could regulate its role as a molecular bridge, linking cellular metabolism, ionic homeostasis, and hormone secretion. The regulation of Na^^+^^/K^^+^^-ATPase activity via ATP1B1 binding may represent a mechanism by which incretin therapies stabilize excitability and secretory function in pancreatic islets, and potentially also in neurons, contributing to their efficacy in T2D and metabolic disease.

**Figure 1:**
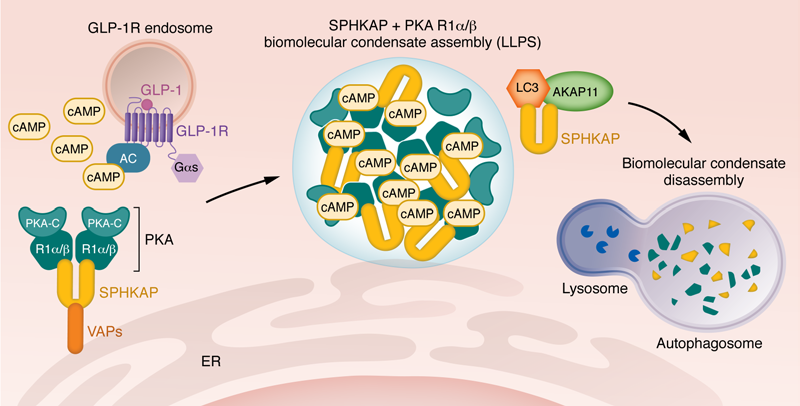
Proposed mechanism of GLP-1R–mediated assembly and disassembly of SPHKAP–PKA RIα/β biomolecular condensates. Active GLP-1Rs are trafficked to endosomes where they engage VAPs to form ER-endosome MCSs, generating local pools of cAMP. VAPs engage SPHKAP to form PKA RIα/β biomolecular condensates upon local PKA activation by GLP-1R–generated cAMP. SPHKAP subsequently recruits AKAP11, triggering LC3-dependent autophagosomal degradation of PKA RIα/β biomolecular condensates. AC, adenylate cyclase; PKA-C, catalytic subunit of PKA; PKA RIα/β, PKA regulatory subunit Iα/β; SPHKAP, sphingosine kinase 1 interactor, AKAP domain–containing; ER, endoplasmic reticulum; AKAP11, A-kinase anchoring protein 11; LC3, microtubule-associated protein 1A/1B-light chain 3; LLPS, liquid-liquid phase separation.

Another notable finding is the association between active GLP-1R and vesicle-associated membrane protein–associated proteins A and B (VAPA/B) that was found in the two independent interactome studies that analyzed β cell–wide GLP-1R interactions (54, 55). VAPA/B are integral endoplasmic reticulum (ER) proteins known for their capacity to establish contacts with multiple membranes by interacting with different tethers (62), and are therefore fundamental constituents of ER membrane contact sites (MCSs) (63). ER MCSs are points of close apposition between the ER and various other organelles that regulate the inter-organelle bidirectional transport of lipids and ions (64). GLP-1R is known to be rapidly internalized and continue to signal from early endosomes (65), consistent with the common interaction observed with the early endosomal marker Rab5A (54, 55). Recent work has further demonstrated that following its internalization, active endosomal GLP-1R engages with VAPA/B to form ER-endosome contacts (54), which also contain the VAPA/B- and PKA RIα/β–binding (66) AKAP sphingosine kinase 1 interactor, AKAP domain–containing (SPHKAP) (42, 43), an AKAP11 paralog involved in the formation of PKA RIα/β biomolecular condensates at ER MCSs (42, 43). Given the association of active GLP-1R with PKA RIα/β rather than RII isoforms (54), it is tempting to hypothesize that GLP-1R might engage VAPA/B and SPHKAP to trigger the assembly of PKA RIα/β condensates at ER MCSs (Figure 1). Highly relevant here is the recently found role of AKAP11 itself in the degradation of VAPA/B- and SPHKAP-associated PKA RIα/β condensates through selective autophagy, via its interaction with the autophagy protein LC3 in neurons (43, 44). Such activity has been shown to be key for the control of synaptic transmission and dopaminergic signaling, with its disruption associated with the development of BD and schizophrenia (67), suggesting that a similar mechanism might be in place in GLP-1R–expressing tissues in the brain and pancreas. Moreover, AKAP11 control of VAPA/B- and SPHKAP-associated PKA RIα/β condensates in the brain has been shown to involve its binding and regulation of GSK3β, a serine/threonine kinase target of the mood-stabilizing drug lithium used to treat BD, and DYRK1A, a tyrosine kinase genetically linked to autism spectrum disorders and implicated in neurodevelopment, neurogenesis, and neurite outgrowth (43, 67). This is highly relevant, as both kinases have been shown to be strongly inhibited by GLP-1R activity in β cells (68), and inhibitors of both are currently under investigation as therapies to improve β cell survival and proliferation (69–71). This suggests that the metabolic improvements associated with these drugs might, at least in part, involve the potentiation of GLP-1R signaling at ER-localized signalosomes.

**Figure 2:**
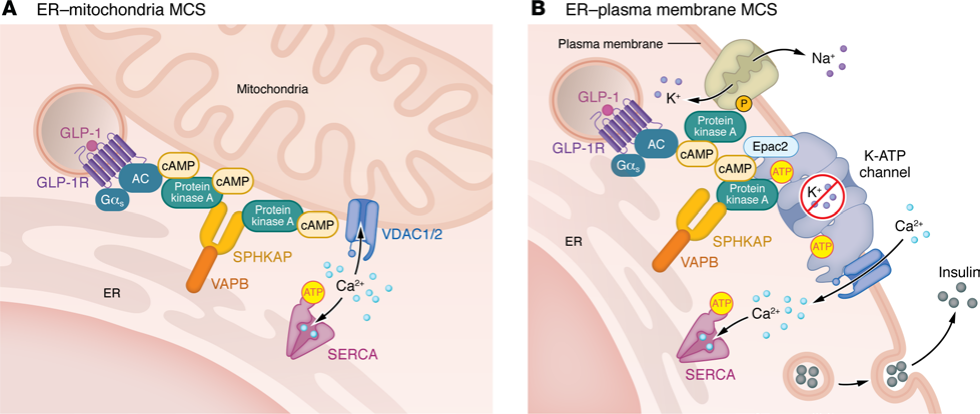
Schematic of the molecular core composition of two predicted GLP-1R signalosomes. (**A**) Endosomal GLP-1R engages with VAPB and SPHKAP at ERMCSs to assemble a local signalosome to regulate ER and mitochondrial Ca²^+^ and ATP fluxes via its interaction with VDAC1/2. (**B**) Endosomal GLP-1R engages with VAPA/B and SPHKAP at ER–plasma membrane contact sites to assemble a local signalosome to regulate localized Ca²^+^ increases coupled to insulin exocytosis via its interaction with SUR1 at K-ATP–LTCC–insulin hotspots.

Engagement of endosomal GLP-1R with VAPB has recently been shown to trigger localized receptor signaling at ER-mitochondria MCSs (ERMCSs), with effects on mitochondrial remodeling and function (54). This signaling also requires a functional SPHKAP-VAPB interaction, and therefore is inferred to involve the establishment of a PKA RIα/β condensate specifically at ERMCSs. Additionally, both β cell–wide interactomes (54, 55) identified the ERMCS-localized voltage-dependent anion channel VDAC1/2 (72) as a common interactor of active GLP-1R. VDAC is known to play important roles in the control of ER-to-mitochondria Ca^^2+^^ transfer (73) and ATP flux (74) both in β cells and in neurons, with T2D defects associated with VDAC1 mistargeting, which causes loss of ATP flux, mitochondrial Ca^^2+^^ dysregulation, and impaired insulin secretion (75). Furthermore, VDAC is part of the mitochondrial permeability transition pore (mPTP). Under normal physiological conditions, the mPTP regulates mitochondrial bioenergetics, but following prolonged opening under increased ROS and ER stress, mPTP dysfunction is linked to β cell death and loss of glucose control in T2D (76). These findings identify the ER-mitochondria junction as a key subcellular location for the control of β cell metabolic coupling, prone to dysregulation under glucolipotoxic conditions. Thus, GLP-1R signaling at ERMCSs (Figure 2A) might restore ER and/or mitochondrial function through the control of Ca^^2+^^ and ATP fluxes, protecting β cells, and potentially also neurons, from energy failure during oxidative and ER stress.

Beyond ERMCSs, a further common β cell interactor identified for active GLP-1R is sulfonylurea receptor 1 (SUR1, encoded by *ABCC8*), the regulatory subunit of the K-ATP channel. SUR1 is a key metabolic sensor known to couple β cell metabolism to insulin secretion, and *ABCC8* mutations are associated with insulin secretion disruptions in T2D (77). Several reports support the existence of K-ATP–channel and L-type calcium channel–containing (LTCC-containing) Ca^^2+^^ microdomains, or hotspots, in the plasma membrane of neuroendocrine cells, with channels preassembled with docked insulin granules, resulting in rapid exocytosis synchronized with membrane depolarization, a process disturbed in T2D (78). GLP-1R, through cAMP generation, modulates the activity of both K-ATP and LTCC, with cAMP acting on the K-ATP channel to increase its sensitivity to ATP via PKA and Epac2 (79). Both SUR1 and the pore subunit of K-ATP, Kir6.2, harbor PKA phosphorylation sites, with GLP-1R–dependent PKA activation leading to SUR1 phosphorylation, lowering its affinity for ADP (80). Similarly, cAMP-dependent activation of Epac2 increases K-ATP sensitivity to ATP via Epac2 dissociation from SUR1 (81). Once dissociated, Epac2 binds Rim2, a small GTPase bound to the LTCC Cav1.2, as well as Piccolo, in a Ca^^2+^^-dependent manner, inducing insulin granule exocytosis through binding of the Epac2-Rim2-Piccolo complex to Rab3 at the insulin granule membrane (82). GLP-1R–induced PKA activity also phosphorylates snapin, enabling its interaction with SNAP-25 to further tether insulin secretory granules to the plasma membrane (83). These data indicate that GLP-1R signaling regulates the function of K-ATP and LTCC channels and local insulin granule mobilization and exocytosis. Together with the above-mentioned interactome results, it is possible that a local GLP-1R cAMP signalosome is assembled at K-ATP–LTCC–insulin hotspots (Figure 2B) to regulate channel function, enabling the spatiotemporally coordinated potentiation of insulin exocytosis by incretins.

Interestingly, AKAP localization to K-ATP–LTCC hotspots has been shown to be essential for the tonic drive exerted by PKA on these channels (84, 85), suggesting that localized PKA phosphorylation events might augment calcium flux (86), and opening the door for the GLP-1R–associated AKAP SPHKAP to play a role in the regulation of GLP-1R signalosome function at these channel hotspots. SPHKAP was previously shown to be involved in neuronal Ca^^2+^^ signaling by localizing PKA RIα/β biomolecular condensates at ER–plasma membrane contact sites via its interaction with VAPA/B, where it influences intracellular Ca^^2+^^ release from ER stores in the vicinity of Cav1.2 (42). Depletion of ER Ca^^2+^^ by thapsigargin, an inhibitor of the sarco/endoplasmic reticulum Ca^^2+^^ ATPase (SERCA) pump, another active GLP-1R interactor (54), renders β cells unresponsive to GLP-1 (87). In contrast, GLP-1R activity triggers Ca^^2+^^-induced Ca^^2+^^ release (CICR) (88), a process where extracellular Ca^^2+^^ influx triggers the release of more Ca^^2+^^ from intracellular stores, amplifying local signals. We can therefore further hypothesize that GLP-1R signalosomes at K-ATP–LTCC–insulin hotspots might engage the ER to trigger local Ca^^2+^^ release at ER–plasma membrane contact sites via the assembly of VAPA/B–SPHKAP–PKA RIα/β condensates. Furthermore, the K-ATP channel is important in neurotransmitter release and dopaminergic signaling, with SUR1 dysregulation contributing to dopaminergic neuron degeneration in Parkinson’s disease (89). Therefore, if the GLP-1R–SUR1 interaction is present in neurons, it might contribute to the protective effects of incretin therapies in neurodegeneration (90). Together with its interaction with the Na^^+^^/K^^+^^-ATPase, GLP-1R’s interaction with SUR1 positions the receptor as a master regulator of localized ion fluxes, potentially fine-tuning electrochemical gradients that underlie excitatory responses and secretory competence (91).

Taken together, the identification of these common GLP-1R interactors suggests the existence of GLP-1R–cAMP–PKA signalosomes at different subcellular locations, potentially engaging specific ER MCSs, reinforcing the view that GLP-1R activity extends beyond the cell surface and is shaped by anchored signaling enzymes within specialized nanodomains.

From a clinical perspective, the embedding of GLP-1R signaling within different ion channel and transporter microenvironments provides a mechanistic basis for the receptor’s ability to potentiate insulin and neurotransmitter release, as well as a link between receptor activity and the control of mitochondrial metabolism and cell survival. These represent key cellular processes required for optimal β cell/islet function and glucose homeostasis, known to be altered in T2D (76, 92), as well as in underlying central nervous system effects on food intake, reward, cognition, and emotional regulation, known to be impacted by GLP-1R activity (93–95).

Understanding the mechanisms behind GLP-1R signal compartmentalization is not only fundamental to increase our knowledge of incretin biology but may also inform the design of next-generation GLP-1R–based therapies or lead to the identification of novel GLP-1R effectors that could be exploited as drug targets with optimized benefits but limited adverse effects.

## Biased incretin therapies and spatiotemporal regulation of GLP-1R

The emergence of biased GLP-1R agonists raises the possibility that ligand-directed effects in G protein versus β-arrestin selectivity, and/or other biased agonism modalities (96), might lead to differences in receptor signal compartmentalization. Ligands that preferentially retain GLP-1R at the plasma membrane might also lead to sustained insulinotropic (97) and weight loss (98) responses by prolonging receptor coupling to Gα__s__, an effect linked to reduced receptor desensitization, despite partial agonism. Conversely, agonists that promote rapid receptor internalization might favor acute endosomal and ER-associated signaling, potentially leading to increased intracellular cAMP generation and ER and/or mitochondrial effects (99). Thus, the spatiotemporal “address” of GLP-1R signalosomes has the potential to become a key determinant of biological outcomes.

Recent evidence highlights how ligand bias can shape the spatiotemporal signaling dynamics of GLP-1R. In β cells, the exendin-4–based, Gα__s__-biased GLP-1R agonist exendin-F1, as well as other Gα__s__-biased agonists with reduced β-arrestin engagement, show diminished acute internalization and interaction with the ER tethering protein VAPB (54). In contrast, dual agonists such as tirzepatide, with balanced targeting of GIPR while biasing GLP-1R toward Gα__s__ (100), may exploit both plasma membrane and internalization-competent conformations by engaging the two incretin receptors at different locations. By allowing for sustained GLP-1R Gα__s__ signaling at the plasma membrane while also engaging β-arrestin scaffolds and endosomal signaling via GIPR, tirzepatide might thereby support further metabolic benefits beyond acute insulin secretion effects (101). Novel small molecule GLP-1R agonists such as danuglipron (102) and orforglipron (103), with distinct engagement of GLP-1R transmembrane domains, also exhibit marked differences in their trafficking and spatiotemporal signaling phenotypes (54), raising the possibility of fine-tuning receptor residencies within particular nanodomains in future GLP-1R drug modalities (104).

From a translational perspective, these findings suggest that biased agonism must be considered not only in terms of downstream effector coupling, but also in relation to receptor engagement with specific subcellular nanodomains. Spatiotemporal bias may help explain why some GLP-1R agonists produce disproportionate benefits on weight loss or cardiovascular endpoints, while others primarily enhance insulin secretion (105, 106). Deliberately targeting receptor localization to specific ER MCSs could represent a new frontier in the design of incretin-based therapies.

## PTMs as regulators of GLP-1R signalosomes

Like many GPCRs, GLP-1R is subject to an array of PTMs that fine-tune receptor function beyond the initial ligand-receptor interaction (107). These covalent modifications act as molecular switches that shape receptor folding, stability, trafficking, and interaction with scaffold proteins, thereby potentially dictating the spatial and temporal properties of GLP-1R signaling. Five major classes of PTMs have been implicated in GLP-1R regulation and are likely to play critical roles in the organization of signaling nanodomains. These include (a) phosphorylation of intracellular loops and the C-terminal tail of the receptor, primarily by GRKs, which regulates β-arrestin recruitment, desensitization, and trafficking (108); (b) glycosylation of extracellular asparagine (Asn) residues, required for correct receptor folding, surface expression, and ligand binding (109); (c) palmitoylation of cytoplasmic cysteine (Cys) residues, tethering the receptor to cholesterol-rich membrane nanodomains, potentially aligning it with ion channels and exocytic machinery (20); (d) ubiquitination, which directs receptors toward lysosomal degradation versus recycling pathways, thereby tuning the duration and localization of GLP-1R signaling at the plasma membrane versus intracellular compartments (110); and (e) SUMOylation, proposed to stabilize receptor-scaffold interactions and modulate endocytosis, potentially contributing to GLP-1R localization at endomembrane signaling hubs (111). Collectively, these PTMs provide a dynamic code that determines how GLP-1R signaling is compartmentalized within the cell.

Phosphorylation is one of the most extensively studied PTMs for GLP-1R, acting as a key regulator of receptor trafficking and signal duration. Recent work has shown that, although GLP-1R internalization occurs independently of β-arrestins, it is strictly dependent on the action of GRKs (16). McNeill et al. demonstrated that deletion of all nonvisual GRKs abolished agonist-mediated receptor internalization, while reintroduction of individual isoforms restored this process, indicating functional redundancy (16). GRKs also differentially shaped β-arrestin engagement, with β-arrestin 1 recruitment strongly dependent on GRK activity, whereas β-arrestin 2 recruitment was less affected. These findings suggest that GRK-mediated phosphorylation functions as a spatial “barcode” for GLP-1R. By modifying specific serine and threonine residues on intracellular domains, GRKs modulate receptor trafficking and determine whether signaling is sustained at the plasma membrane, redistributed to endosomes, or, potentially, coupled to ER MCSs. In this way, phosphorylation serves as an early layer of code, translating ligand binding into spatiotemporally distinct signaling outcomes.

Glycosylation is a critical PTM of GLP-1R, essential for its proper folding, trafficking, and ligand recognition. Huang et al. (112) identified three N-linked glycosylation sites (Asn^^63^^, Asn^^82^^, and Asn^^115^^) within the large extracellular N-terminal domain of the receptor. Mutational analysis showed that removal of these sites disrupts receptor glycosylation and reduces its cell surface expression, severely impairing GLP-1R signaling. Importantly, glycosylation-deficient mutants exhibited markedly reduced GLP-1 binding, highlighting the dual role of this modification in both receptor stability and ligand engagement. From a spatial perspective, glycosylation ensures efficient delivery of correctly folded receptors to the plasma membrane, maintaining the density of signaling-competent GLP-1Rs. Thus, N-linked glycans act as a quality-control checkpoint that both safeguards ligand responsiveness and indirectly defines the geography of receptor signaling, embedding glycosylation into the GLP-1R signaling code.

Palmitoylation represents another key PTM of the GLP-1R, dynamically regulating its membrane organization and signaling competence (113). Both Buenaventura et al. (20) and Naglekar et al. (114) investigated a palmitoylation site at Cys^^438^^ in the C-terminal tail of GLP-1R, where reversible acylation promotes receptor clustering into cholesterol-rich membrane nanodomains. Mutation of this site reduced agonist-induced palmitoylation and receptor-cholesterol interactions, and impaired both G protein coupling and β-arrestin recruitment, underscoring its central role in receptor conformational flexibility and signal propagation (20). From a spatial perspective, palmitoylation acts as a molecular switch that orders GLP-1R into lipid raft–like microdomains where it can cocluster with membrane proteins such as the Na^^+^^/K^^+^^-ATPase and the K-ATP channels. By positioning the receptor alongside these ion channels and transporters, palmitoylation ensures efficient coupling of GLP-1R cAMP signaling to changes in membrane excitability and exocytosis. Thus, beyond simply anchoring the receptor in cholesterol-rich regions, palmitoylation defines the nanodomain geography that links GLP-1R activation to the previously discussed channel-based interactome.

Ubiquitination is an important determinant of GLP-1R trafficking fate. Bitsi et al. (115) showed that β-arrestin 2 recruitment not only mediates acute GLP-1R desensitization but also supports receptor recycling, with its loss leading to altered GLP-1R trafficking and divergent insulin secretory responses. Mechanistically, β-arrestins are known to recruit E3 ligases such as NEDD4, coupling receptor ubiquitination to endosomal sorting (116). In this framework, deubiquitination promotes receptor recycling, whereas persistent ubiquitination drives lysosomal degradation, thereby defining receptor longevity. Ubiquitination therefore acts as a trafficking “code” that balances GLP-1R residency at the plasma membrane versus intracellular signaling platforms, ensuring the temporal fidelity of insulin secretion and preserving the receptor’s capacity to reengage with intracellular signalosomes such as those localized at ERMCSs.

SUMOylation provides further spatiotemporal regulation by shifting the receptor away from the plasma membrane and retaining it in intracellular compartments under hyperglycemic conditions (117), a process dependent on PKA phosphorylation of the receptor at Ser^^301^^, as shown in Rajan et al. (118). GLP-1R SUMOylation therefore reduces its availability at the plasma membrane, dampening acute cAMP signaling under high glucose exposure. Of note, the interactome analysis performed by Austin et al. (54) showed that GLP-1R is constitutively SUMOylated and becomes de-SUMOylated upon exendin-4 stimulation, suggesting that agonist binding actively relieves this constraint to restore receptor accessibility within signaling nanodomains. As SUMOylation is increasingly recognized as a regulator of biomolecular condensate dynamics (119), it will be important to determine whether GLP-1R SUMOylation/de-SUMOylation also influences condensate assembly at intracellular signaling hubs.

Taken together, these different PTMs constitute a spatiotemporal “code” that dictates the itinerary and duration of GLP-1R signaling. An important emerging idea is that this PTM code may not only direct receptor localization to discrete nanodomains but also influence the assembly of higher-order biomolecular condensates (49). This could occur either by directly promoting receptor aggregation and LLPS, or indirectly by dictating receptor trafficking to specific subcellular locations such as ER MCSs or promoting receptor recruitment of effectors such as β-arrestins, capable of undergoing LLPS to form condensates that regulate GPCR internalization and downstream signaling (48). This framework provides a mechanistic link between receptor PTMs, spatiotemporal signaling organization, and biomolecular condensate formation.

Clinically, if GLP-1R signaling occurs via biomolecular condensates, defects in condensate assembly, stability, or dissolution — whether through altered receptor PTMs or via defects in regulatory factors that affect this process — may either blunt or exacerbate endogenous GLP-1R responses. This could lead to metabolic or neurological disturbances, and contribute to variable therapeutic responses to GLP-1R agonists, highlighting condensate integrity as a potential target to improve incretin responses.

## Final comments

The complexity of the GLP-1R spatiotemporal signaling architecture is compounded by its engagement within a wide network of protein-protein interactions that modulate its localization, desensitization, and signaling bias. Beyond the interactome factors highlighted in this Review, GLP-1R is known to interact with components of clathrin-coated pits such as the clathrin adaptor AP2 (54, 57), small GTPases of the endocytic pathway such as Rab5, Rab7, and Rab11 (54, 55, 120), and specific effectors such as GRKs (121), β-arrestins (17), and RAMPs (14), in isoform-specific ways. GLP-1R also directly interacts with different membrane lipid species (122, 123), with both protein and lipid interactions refining receptor signaling outputs across different tissues and contexts.

While whole cell GLP-1R interactome mapping in different cell types has begun to illuminate this diversity, the field is now evolving toward spatiotemporally resolved approaches. These include methods such as the GLP-1–APEX2 proximity labeling developed by Dang et al. (56), which enabled dynamic mapping of plasma membrane GLP-1R interactors at multiple time points and revealed four distinct temporal clusters. Another advance is the use of split-TurboID–based proximity proteomics approaches (124, 125), which can potentially resolve the local composition of individual GLP-1R signalosomes. Despite the growing recognition of the importance of spatiotemporally organized nanodomains, our current understanding of GLP-1R signaling remains largely limited to static or bulk measurements that fail to capture its dynamic and compartmentalized nature. Dissecting the signaling architecture of this receptor will require the integration of advanced methodologies capable of resolving interactions and signaling events with high spatial and temporal fidelity. Emerging tools such as real-time biosensors and optogenetic approaches, super-resolution microscopy, including in situ structural biology methods such as cryo-electron tomography (cryo-ET) and cryo-correlative light and electron microscopy (cryo-CLEM), as well as spatial proteomics and the above-mentioned proximity labeling approaches offer unprecedented opportunities to map the assembly, localization, and kinetics of individual receptor-associated signaling complexes. Coupled with computational modeling and spatial transcriptomics, these approaches hold the promise to reveal how distinct nanodomains orchestrate context-specific endogenous responses as well as pharmacological responses to current and novel GLP-1R agonists. Moving beyond traditional linear signaling paradigms, the next frontier lies in decoding the organized and dynamic architecture that underpins receptor function across multiple physiological and pathological contexts. As spatially resolved approaches continue to evolve, such insights promise to refine our understanding of the organization of signaling of GLP-1R and other members of this family of class B1 GPCRs, uncovering new opportunities for therapeutic intervention.

In summary, GLP-1R functions not as a surface-level switch, but as an architect of subcellular signaling nanodomains that integrate Ca^^2+^^ and cAMP dynamics through time-sensitive, organelle-localized protein-protein and protein-lipid interactions. This framework fundamentally redefines how we understand receptor outputs, from linear pathways to spatiotemporally encoded signalosomes.

## Figures and Tables

**Table 1:**
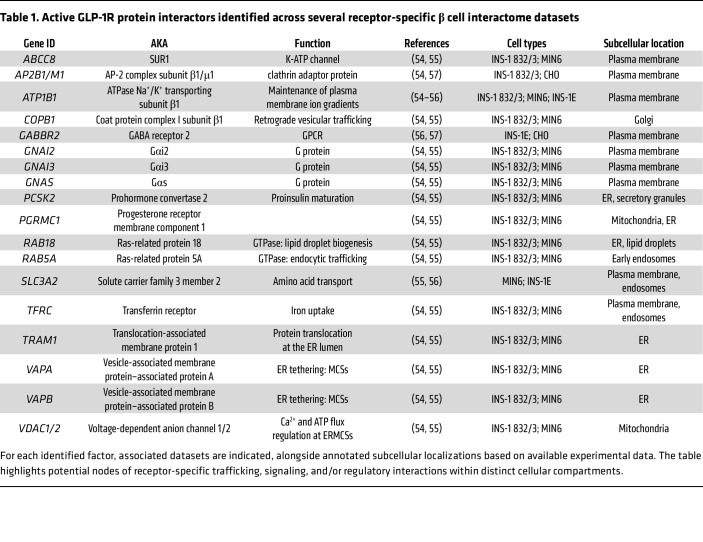
Active GLP-1R protein interactors identified across several receptor-specific β cell interactome datasets

## Version 1 01/16/2026

Electronic publication
